# Anti-Obesity Effects of Polymethoxyflavone-Rich Fraction from Jinkyool (*Citrus sunki* Hort. ex Tanaka) Leaf on Obese Mice Induced by High-Fat Diet

**DOI:** 10.3390/nu14040865

**Published:** 2022-02-18

**Authors:** Yeong-Jun Jin, Mi-Gyeong Jang, Jae-Won Kim, Songyee Baek, Hee-Chul Ko, Sung-Pyo Hur, Se-Jae Kim

**Affiliations:** 1 Department of Biology, Jeju National University, Jeju 63243, Korea; y2j-1004@hanmail.net; 2 Biotech Regional Innovation Center, Jeju Nation University, Jeju 63423, Korea; mkjang@jejunu.ac.kr (M.-G.J.); kjw8839@jejunu.ac.kr (J.-W.K.); summerbee@jejunu.ac.kr (S.B.); 3 Jeju Institute of Korean Medicine, Jeju 63309, Korea; fly1007@jikom.or.kr; 4 Jeju International Marine Science Research & Logistics Center, Korea Institute of Ocean Science & Technology, Gujwa, Jeju 63349, Korea; hursp@kiost.ac.kr

**Keywords:** *Citrus sunki*, polymethoxyflavones, anti-obesity, high-fat diet, C57BL/6, fatty-acid oxidation, lipolysis

## Abstract

Polymethoxyflavones (PMFs) are flavonoids exclusively found in certain citrus fruits and have been reported to be beneficial to human health. Most studies have been conducted with PMFs isolated from citrus peels, while there is no study on PMFs isolated from leaves. In this study, we prepared a PMF-rich fraction (PRF) from the leaves of *Citrus sunki* Hort ex. Tanaka (Jinkyool) and investigated whether the PRF could improve metabolic decline in obese mice induced by a high-fat diet (HFD) for 5 weeks. The HFD-induced obese mice were assigned into HFD, OR (HFD + orlistat at 15.6 mg/kg of body weight/day), and PRF (HFD + 50, 100, and 200 mg/kg of body weight/day) groups. Orlistat and PRF were orally administered for 5 weeks. At the end of the experiment, the serum biochemical parameters, histology, and gene expression profiles in the tissues of each group were analyzed. The body weight gain of the obese mice was significantly reduced after orlistat and PRF administration for 5 weeks. PRF effectively improved HFD-induced insulin resistance and dyslipidemia. Histological analysis in the liver demonstrated that PRF decreased adipocyte size and potentially improved the liver function, as it inhibited the incidence of fatty liver. PRF activated AMP-activated protein kinase (AMPK), acetyl-CoA carboxylase (ACC), and hormone-sensitive lipase (HSL) in HFD-induced obese mice. Moreover, liver transcriptome analysis revealed that PRF administration enriched genes mainly related to fatty-acid metabolism and immune responses. Overall, these results suggest that the PRF exerted an anti-obesity effect via the modulation of lipid metabolism.

## 1. Introduction

Obesity is a complex, multifactorial disorder that is primarily caused by an imbalance between energy uptake and expenditure. The prevalence of obesity is increasing worldwide and its associated metabolic disorders are a major public health concern [1]. Moreover, obesity contributes to chronic diseases such as type 2 diabetes, nonalcoholic fatty liver disease, hypertension, heart disease, osteoarthritis, and obstructive sleep apnea, and increases the risk of several cancers and other diseases [2,3,4,5]. In addition, people living with obesity often experience mental health issues alongside different degrees of functional limitations, i.e., obesity-related disability, and they suffer from social bias, prejudice, and discrimination [6].

Currently, multiple therapeutic methods with which to treat obesity are available, including strengthening exercises, diet control, behavioral changes, surgical treatment, and pharmacotherapy. However, these therapeutic options have the following problems: their time requirements and economic costs are high, many have side effects, and the weight is easily regained after stopping the drugs [7,8,9]. Epidemiological studies have revealed a positive correlation between a high intake of flavonoid-rich foods and the incidence of metabolic syndrome [10,11]. Therefore, recently, bioactive compounds from plant extracts, including citrus flavonoids, have attracted more attention for curing obesity. Citrus flavonoids derived from citrus plants are polyphenolic compounds, and they are divided into three major subgroups: flavanones, flavone glycosides, and polymethoxyflavones (PMFs). PMFs are flavones with two or more methoxyl groups, which exhibit various physiological and pharmacological bioactivities. In particular, PMFs are of great interest due to their beneficial properties, such as anti-obesity, anticancer, and anti-inflammatory activities, both in vitro and in vivo [12,13,14,15,16,17].

*Citrus sunki* Hort ex. Tanaka (commonly known as Jinkyool) is a native citrus plant cultivated in Jeju-do, Korea, and its peel extract is known to contain a large amount of PMFs with various biological activities [18,19,20]. PMFs have been found in the seeds, leaves, juice, stems, and peels of citrus. However, most studies have been conducted on the flavedo and peels of citrus fruits, and there is no study on PMFs isolated from citrus leaves. In this study, we prepared a PMF-rich fraction (PRF) from *C. sunki* leaf and evaluated its efficacy as an anti-obesity ingredient for obese mice whose obesity was induced by a high-fat diet (HFD).

## 2. Materials and Methods

### 2.1. Plant Material and Preparation of Polymethoxyflavone-Rich Fraction (PRF)

**Figure 1:**
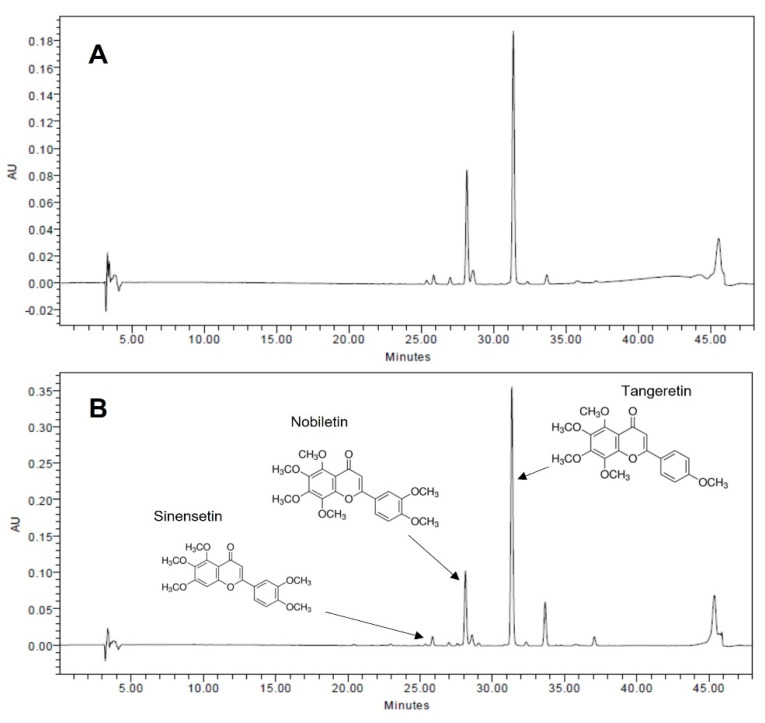
HPLC chromatograms of PMF-rich fraction (PRF) from fruits (**A**) and leaves of Jinkyool (*Citrus sunki* Hort ex. Tanaka) (**B**). Tangeretin, nobiletin, and sinensetin were the three major PMFs contained in PRF.

The Jinkyool fruits and leaves used in this study were collected in Jeju-do, Korea, in February 2021. The collected fruits and leaves were washed immediately, dried at 60 °C for 24 h, and then powdered. The PMF-rich fraction (PRF) was made according to the method of Ko et al. [20]. Briefly, the pulverized powder was extracted with hot water at 100 °C for 5 h. The hot water extract was mixed with hexane in a 1:1 ratio; the hexane layer was recovered, concentrated, lyophilized, and stored at −20 °C until it was used in experiments. The contents of three major PMFs (nobiletin, tangeretin, and sinensetin) in the hexane fraction were quantified using a high-performance liquid chromatography (HPLC) system comprising two pumps, an autosampler, a column oven, and a PDA detector (Waters 2695 Alliance system). The instrument control and data acquisition were accomplished using the Empower software (version 2.0; Waters) and a Sunfire C18 column (4.6 × 250 mm, 5 µm; Waters), monitored at 330 nm. The fraction from whole fruits contained 22.03 mg/g of PMFs (nobiletin, 8.07 mg/g, and tangeretin, 13.96 mg/g), while that of the leaves contained 67.25 mg/g of PMFs (sinensetin, 1.10 mg/g; nobiletin, 15.81 mg/g; and tangeretin, 50.34 mg/g) (Figure 1). Thus, the hexane fraction from the leaves was designated as the PMF-rich fraction (PRF) and used in the subsequent experiment.

### 2.2. Animals and Experimental Design

Four-week-old male C57BL/6J mice were obtained from Cronex Inc. (Gyeonggi-do, Korea). The animals were acclimated for 1 week by housing in appropriate cages under a 12 h/12 h light/dark cycle at room temperature (23 ± 2 °C) and 55% ± 5% relative humidity. After the acclimation period, the animals were fed a normal diet consisting of 14% fat, 21% protein, and 65% carbohydrate (total energy 4.04 kcal/g) (ND, LabDiel 5L79, Orient Bio. Inc. Seongnam-si, Gyeonggi-do, Korea) or a high-fat-diet (HFD), consisting of 60% fat, 20% protein, and 20% carbohydrate (total energy 5.24 kcal/g) (D12492, Research Diets, Inc. New Brunswick, NJ, USA) to induce obesity, for 5 weeks [21]. Then, the HFD-induced obese mice were assigned into HFD, OR (HFD + orlistat at 15.6 mg/kg of body weight/day), and PRF (HFD + 50, 100, and 200 mg/kg of body weight/day) groups. Orlistat and PRF were orally administered at a given dose daily for 5 weeks. The concentration of orlistat was determined by referring to previous reports [22,23]; the drugs were diluted in 0.5% carboxymethyl cellulose (CMC) and administered once daily.

### 2.3. Oral Glucose Tolerance Test (OGTT)

The OGTT was performed at the final experimental day. The experimental animals fasted for at least 8 h, and then glucose (2 g/kg of body weight) was administered orally. After glucose administration, rat tail vein blood was taken at 30, 60, 90, and 120 min to measure the blood glucose level. The HOMA-IR index was assessed by multiplying the fasting serum insulin and fasting serum glucose, and then dividing the result by a constant number [24]: HOMA-IR = (fasting glucose (mg/dL) × fasting insulin (mU/L))/405.

### 2.4. Collection of Serum and Tissue Samples

Blood was taken from the hearts of mice that had fasted for 8 h and been anaesthetized with isoflurane from Vspharm (Gyeonggi, South Korea). The blood was allowed to stand at room temperature for 5 h or more to coagulate, and the coagulated blood was centrifuged at 3000× *g* for 20 min to separate the serum. The separated serum was stored at −70 °C until blood analysis. The liver, adipose tissue, spleen, and kidneys were extracted, weighed, and stored in liquid nitrogen for analysis.

### 2.5. Biochemical Parameter Assays

The serum triglyceride (TG), total cholesterol (TC), high-density lipoprotein cholesterol (HDL-C), and low-density lipoprotein cholesterol (LDL-C) levels were determined using commercial kits (DoGenBio, Seoul, South Korea). The serum glutamic oxaloacetic transaminase (GOT) and glutamic pyruvic transaminase (GPT) levels were determined using commercial kits from Asan Pharm (Gyeonggi, South Korea), according to the manufacturer’s protocol. The insulin levels were determined using rat insulin ELISA kits (Mercodia, Uppsala, Sweden).

### 2.6. Histological Analysis

The adipose and liver tissues were extracted and fixed in 4% paraformaldehyde for 48 h. The tissues were dehydrated with an ethanol series, embedded in paraffin wax, and sectioned into 7 μm-thick slices. After deparaffinization and rehydration, the tissue sections were stained with hematoxylin and eosin (H&E). Then, the stained sections were dehydrated and sealed with Canada balsam (Junsei, Tokyo, Japan), and observed under a microscope (BX-51, Olympus, Tokyo, Japan).

### 2.7. Western Blot Analysis

Western blot analysis was performed according to a previous report [25]. The adipose tissue was homogenized in cold lysis buffer (1 × RIPA buffer, 1 mM phenylmethylsulfonyl fluoride, 1 mM Na_3_VO_4_, 1 mM NaF, 1 μg/mL aprotinin, 1 μg/mL pepstatin, and 1 μg/mL leupeptin) and gathered by centrifugation at 14,000 rpm for 20 mim. The lysate protein concentrations were quantified using a protein assay kit (Bio-Rad, Hercules, CA, USA). The proteins were separated using electrophoresis on sodium dodecyl sulfate–polyacrylamide gels and then moved to polyvinylidene fluoride membranes. The membranes were blocked with 5% (*w*/*v*) skim milk and 0.1% (*v*/*v*) Tween-20 in Tris-buffered saline (TBST). The membranes were incubated with primary antibodies (dilution 1:2000–10,000) against β-actin (Santa Cruz, CA, USA), phospho-AMP-activated protein kinase (pAMPK) and AMPK (Cell Signaling technology, Beverly, MA, USA), phosphor-acetyl-CoA carboxylase (pACC) and ACC (Cell Signaling Technology), and phosphor-hormone-sensitive lipase (pHSL) (Cell Signaling Technology) overnight at 4 °C. The membranes were cleaned with 0.1% TBST and then reacted at room temperature for 1 h with a peroxidase-conjugated secondary antibody (dilution 1:5000–10,000) (Vector Laboratories, Burlingame, CA, USA). The membranes were cleaned with 0.1% TBST; then, the proteins were identified using the Westar ETA C 2.0 substrate (Cyanagen, Bologna, Italy).

### 2.8. RNA-Seq Library Preparation and Sequencing

Liver tissue samples were homogenized to extract the RNA, and the total RNA was prepared using TRIzol reagent (Invitrogen, MA, USA). The RNA quality was evaluated using an Agilent 2100 Bioanalyzer (Agilent Technologies, Amstelveen, The Netherlands, USA), and RNA quantification was executed by an ND-2000 Spectrophotometer (Thermo Inc., DE, USA). Libraries were made from total RNA using the NEBNext Ultra II Directional RNA-Seq Kit (NEW ENGLAND BioLabs, Inc., Hitchin, UK). The mRNA was isolated using the Poly(A) RNA Selection Kit (LEXOGEN, Inc., Vienna, Austria), and used for the cDNA synthesis and shearing according to the manufacturer’s instructions. Indexing was performed using the Illumina indices 1–12. The enrichment step was performed using PCR. Subsequently, the libraries were verified using the TapeStation HS D1000 Screen Tape (Agilent Technologies, Amstelveen, The Netherlands) to assess the mean fragment size. Quantification was conducted using the library quantification kit with a StepOne Real-Time PCR System (Life Technologies, Inc., Carlsbad, CA, USA). High-throughput sequencing was achieved as paired-end 100 sequencing using a NovaSeq 6000 (Illumina, Inc., San Diego, CA, USA).

### 2.9. Data Analysis

Quality control of the raw sequencing data was achieved using FastQC [26]. Adapters and low-quality reads (<Q20) were withdrawn using FASTX_Trimmer [27] and BBMap [28]. Then, the trimmed reads were mapped to the reference genome using TopHat [29]. The RC (read count) data were processed according to the FPKM + geometric normalization method using EdgeR within R [30]. FPKM (fragments per kb per million reads) values were estimated using Cufflinks [31]. Data mining and graphic visualization were accomplished using ExDEGA (Ebiogen Inc., Seoul, Korea).

### 2.10. Statistical Analysis

All the statistical analyses were performed using SPSS version 21.0 for Windows (SPSS; Chicago, IL, USA). All the data are presented as means ± standard deviations (SDs). Differences between groups were examined by Duncan multiple test in one-way analysis of variance (ANOVA). A *p*-value < 0.05 was considered to indicate statistical significance.

## 3. Results

### 3.1. Effects of PRF on the Body Weight (BW) in HFD-Induced Obese Mice

It was confirmed that obesity was induced in mice fed a high-fat diet for 5 weeks by the considerable increase in body weight compared to the normal-diet group (the initial weights are shown in Table 1). The HFD-induced obese mice were further divided into five groups and administered orlistat or PRF orally for 5 weeks. The OR and PRF groups exhibited a significant decrease in body weight compared to the high-fat diet group (final weights in Table 1). In particular, PRF decreased the weight gain in a dose-dependent manner. During the feeding period, the food intake was higher in the ND group than in the HFD group, but it did not differ significantly among the HFD, OR, and three PRF groups. The weights of the liver, epididymal fat, kidney, and spleen were higher in the HFD group compared with the ND group. However, the weights of the liver were significantly decreased in the orlistat and PRF groups compared with the HFD group. In particular, the weights of white adipose tissue (epididymal fat) and the spleen were effectively decreased in the PRF group, indicating that the PRF modulated lipids and the immune response. There were no significant differences in kidney weights among the HFD, OR, PRF 50, PRF 100, and PRF 200 groups. Similarly to orlistat, PRF substantially reduced the body weight gain in HFD-induced obese mice; however, we did not detect any sign of PRF-induced toxicity.

### 3.2. Effects of PRF on Serum Lipid Profiles in HFD-Induced Obese Mice

**Figure 2:**
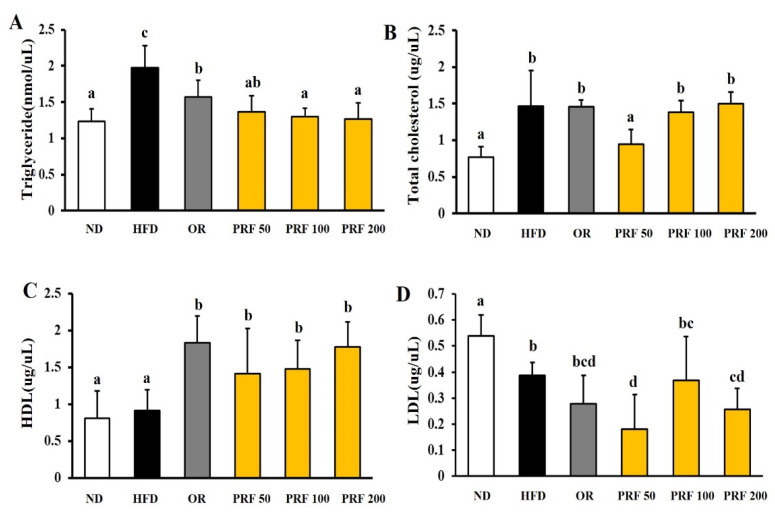
Effect of PRF on serum lipid profiles in HFD-induced obese mice. HFD-induced obese mice were orally administered the drug under an HFD for a further 5 weeks. (**A**) Triglyceride levels, (**B**) total cholesterol levels, (**C**) HDL-cholesterol levels, (**D**) LDL-cholesterol levels. ND, normal diet; HFD, high-fat diet; OR, HFD + 15.6 mg of orlistat; PRF 50, PRF 100, and PRF 200, HFD + 50, 100, and 200 mg of PRF, respectively. The results are expressed as the means ± SDs (*n* = 7). The values with different letters (a–d) are significantly different (*p* < 0.05) from each other according to one-way ANOVA and Duncan’s multiple-range test.

At the end of the 10-week experimental period, we compared the serum lipid profiles of each group. The serum TG and TC levels were higher in the HFD group than in the ND group, but there was no difference in HDL levels between the two groups. The LDL level was rather high in ND group (Figure 2). Orlistat or PRF administration for 5 weeks to HFD-induced obese mice significantly improved the serum TG and HDL levels (Figure 2A,C). However, there was no difference in TC or LDL levels in the HFD, OR, PRF 100, and PRF 200 groups, in contrast to the PRF 50 group.

### 3.3. Effects of PRF on the Insulin Resistance in HFD-Induced Obese Mice

**Figure 3:**
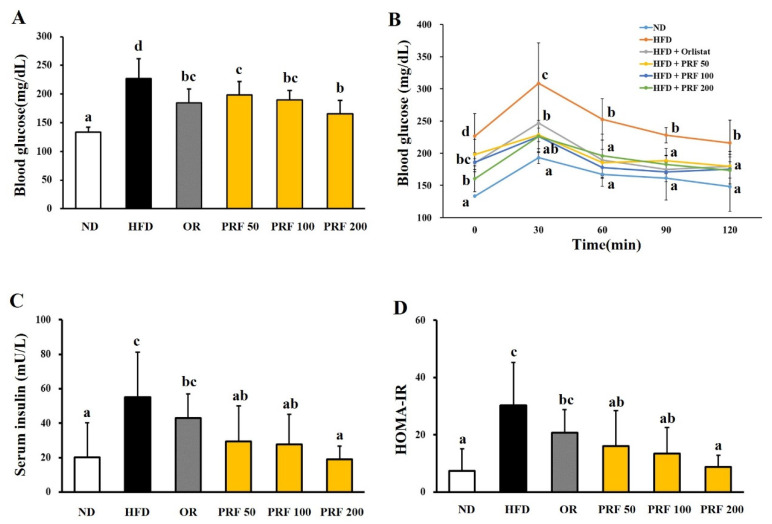
Effect of PRF on glucose homeostasis in HFD-induced obese mice. The mice were fasted for 8 h before measuring blood glucose. (**A**) Fasting blood glucose levels; (**B**) oral glucose tolerance test (OGTT); (**C**) serum insulin levels; (**D**) HOMA-IR index. ND, normal diet; HFD, high-fat diet; OR, HFD + 15.6 mg of orlistat; PRF 50, PRF 100, and PRF 200, HFD + 50, 100, and 200 mg of PRF, respectively. The results are expressed as the means ± SDs (*n* = 7). The values with different letters (a–d) are significantly different (*p* < 0.05) from each other according to one-way ANOVA and Duncan’s multiple-range test.

The fasting blood glucose levels were higher in the HFD group than the ND group, indicating that the HFD induced hyperglycemia in this animal model. The OR and three PRF groups showed attenuation of HFD-induced hyperglycemia (Figure 3A). The oral glucose tolerance test showed that HFD-induced glucose tolerance was significantly improved by OR and PRF administration (Figure 3B). Moreover, the serum insulin level in HFD-induced obese mice was dramatically reduced by OR and PRF (Figure 3C). PRF effectively reduced the homeostasis model assessment insulin resistance (HOMA-IR) index in a dose-dependent manner (Figure 3D).

### 3.4. Effects of PRF on the Hypertrophy of Adipocytes in HFD-Induced Obese Mice

**Figure 4:**
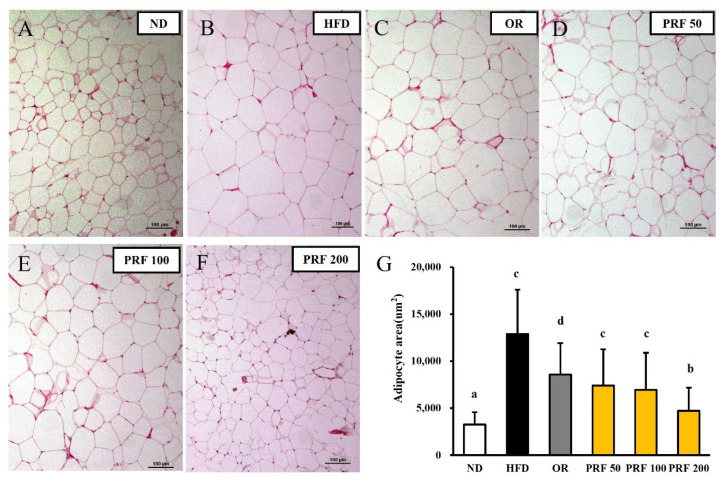
Effect of PRF on the size of epididymal adipocytes in HFD-induced obese mice. The sections of the epididymal fat tissues of ND (**A**), HFD (**B**), OR (**C**), PRF 50 (**D**), PRF 100 (**E**), and PRF 200 (**F**) were stained with H&E (100×). Scale bar = 100 µm. (**G**) The epididymal adipocyte sizes of each group. ND, normal diet; HFD, high-fat diet; OR, HFD + 15.6 mg of orlistat; PRF 50, PRF 100, and PRF 200, HFD + 50, 100, and 200 mg of PRF, respectively. The results are expressed as the means ± SDs (*n* = 7). The values with different letters (a–d) are significantly different (*p* < 0.05) from each other according to one-way ANOVA and Duncan’s multiple-range test.

After 5 weeks of oral administration, the morphometric analysis of epididymal adipose tissue showed a difference among the experimental groups. The sizes of the epididymal adipocytes in each group are shown in Figure 4. The sizes of the adipocytes were appreciably larger in the HFD group than in the ND group. The sizes of visceral adipocytes were considerably decreased in the OR and PRF groups compared with the HFD group. This indicates that weight gain is a result of the accumulation of triglycerides in fat cells in white adipose tissue (WAT) and the expansion of the adipose tissue. In line with the action of orlistat (an anti-obesity drug), PRF ameliorated the hypertrophy of WAT adipocytes in HFD-induced obese mice.

### 3.5. Effects of PRF on Fatty-Acid Oxidation and Lipolysis in HFD-Induced Obese Mice

**Figure 5:**
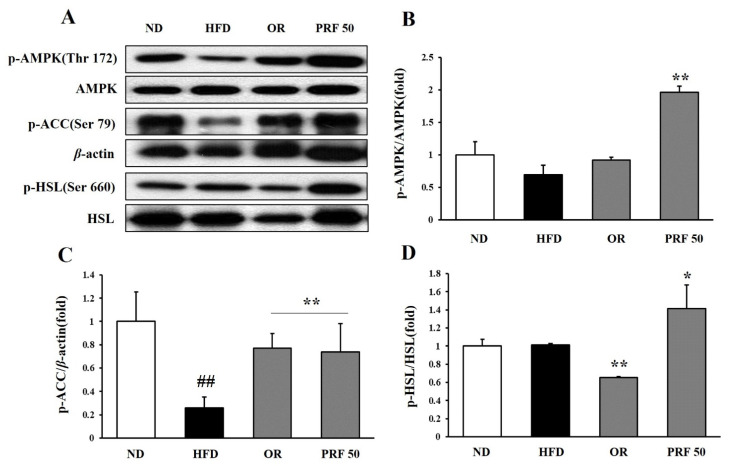
Effect of PRF on the regulation of lipid-metabolism-related protein expression in epididymal adipose tissue in HFD-induced obese mice. The expression of p-AMPK, AMPK, p-ACC, p-HSL, HSL, and β-actin was analyzed by Western blot analysis (**A**), and the intensity of each band was measured using ImageJ. The relative intensity of p-AMPK/AMPK (**B**), p-ACC/β-actin (**C**), and p-HSL/HSL (**D**). The results are expressed as the means ± SDs (*n* = 3; ^##^ *p* < 0.01 compared to ND; * *p* < 0.05, ** *p* < 0.01 compared to HFD). ND, normal diet; HFD, high-fat diet; OR, HFD + 15.6 mg of orlistat; PRF 50, PRF 100, and PRF 200, HFD + 50, 100, and 200 mg of PRF, respectively.

To explore the underlying molecular mechanisms of the anti-obesity effects of PRF administration, we investigated the expression of epididymal adipose proteins among the groups. PRF administration dramatically enhanced the phosphorylation of AMP-activated protein kinase (AMPK), acetyl-CoA carboxylase (ACC), and hormone-sensitive lipase (HSL) in HFD-induced obese mice (Figure 5). These results suggest that PRF exerted its anti-obesity effects by promoting fatty-acid oxidation and lipid lysis in HFD-induced obese mice.

### 3.6. Effects of PRF on Liver Function in HFD-Induced Obese Mice

**Figure 6:**
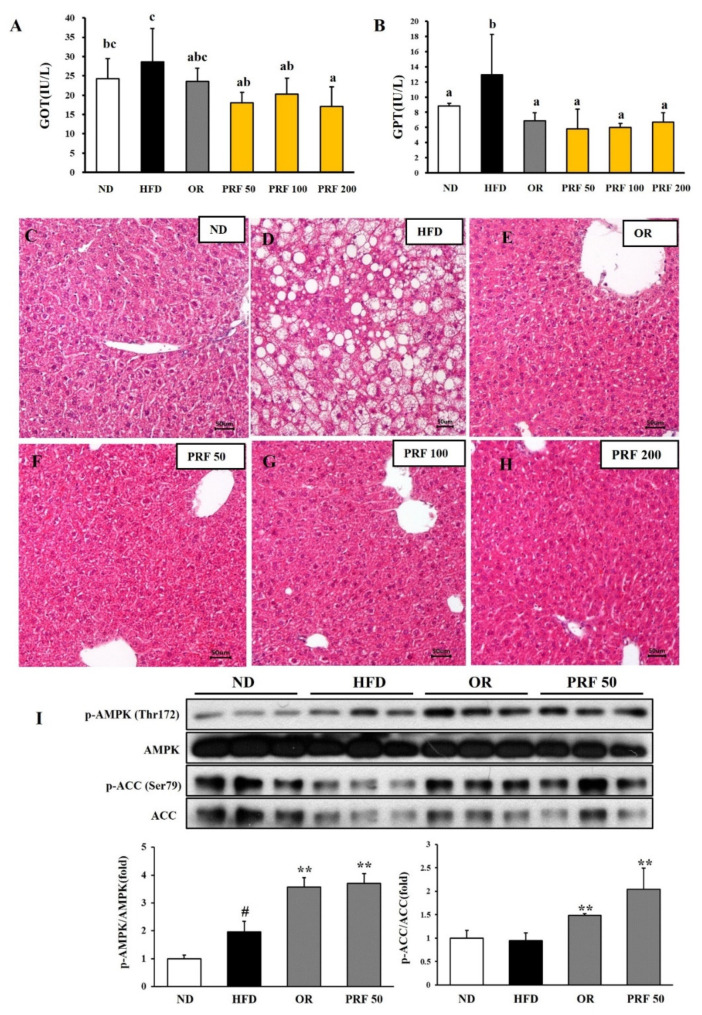
Effect of PRF on liver function in HFD-induced obese mice. (**A**) GOT levels; (**B**) GPT levels. The results are expressed as the means ± SDs (*n* = 7). The values with different letters (a–c) are significantly different (*p* < 0.05) from each other according to one-way ANOVA and Duncan’s multiple-range test. The liver sections for ND (**C**), HFD (**D**), OR (**E**), PRF 50 (**F**), PRF 100 (**G**), and PRF 200 (**H**) stained with H&E (200×). Scale bar = 50 µm. (**I**) Western blot of AMPK and ACC. The results are expressed as the means ± SDs (*n* = 3; ^#^ *p* < 0.05 compared to ND; ** *p* < 0.01 compared to HFD). ND, normal diet; HFD, high-fat diet; OR, HFD + 15.6 mg of orlistat; PRF 50, PRF 100, and PRF 200, HFD + 50, 100, and 200 mg of PRF, respectively.

The serum glutamic oxaloacetic transaminase (GOT) and glutamic pyruvic transaminase (GPT) levels, which are delicate clinical indicators of liver damage, were elevated in the HFD group compared with the ND group. However, OR and PRF administration effectively reduced the GOT/GPT levels in HFD-induced obese mice (Figure 6A,B), indicating their preventive effects on the liver damage induced by the HFD. Consistent with this result, the histological analysis revealed that the HFD group progressed to a fatty liver by accumulating larger lipid droplets. However, OR and PRF administration significantly ameliorated the HFD-induced fatty liver (Figure 6C–H). Since AMPK activation is linked with metabolic improvement, we compared the phosphorylation of AMPK and ACC among the experimental groups. As shown in Figure 6I, orlistat and PRF administration activated AMPK and ACC, indicating that AMPK played a vital role in mediating the beneficial effect of PRF in HFD-induced obese mice.

### 3.7. Effects of PRF on the Liver Transcriptome in HFD-Induced Obese Mice

**Figure 7:**
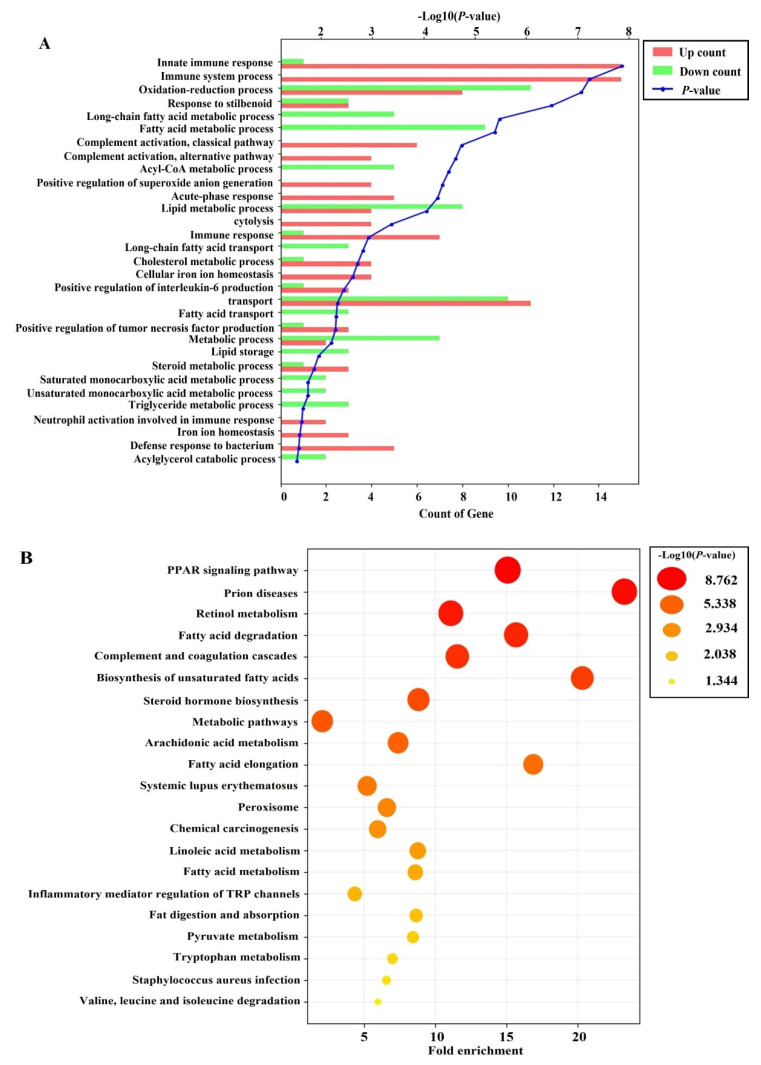
Effects of PRF administration on the liver transcriptome in HFD-induced obese mice. (**A**) Gene Ontology (GO) analysis using differentially expressed genes (DEGs) between the PRF 50 and HFD groups. Top 30 terms within the biological process category of GO function analysis. (**B**) Kyoto Encyclopedia of Genes and Genomes (KEGG) pathway analysis using DEGs between the PRF 50 and HFD groups. Top 20 terms among KEGG pathways. A *p*-value < 0.05 was considered to reflect statistical significance.

To explore how PRF administration affected whole-gene expression in HFD-induced obese mice, we further analyzed the liver transcriptome profiles using RNA-Seq in the HFD and PRF 50 groups (*n* = 3). To identify the differentially expressed genes (DEGs) between the HFD and PRF 50 groups, the transcriptomic profiles were compared. Among 23,183 genes, 117 DEGs were identified (Table S1), comprising 61 upregulated genes and 56 downregulated genes in the PRF vs. HFD groups, respectively (*p* < 0.05; fold change >2). To elucidate the signatures of the DEGs, Gene Ontology (GO) and Kyoto Encyclopedia of Genes and Genomes (KEGG) pathways were analyzed. PRF administration mainly affected functions in the biological process category of GO terms, such as fatty-acid metabolic process, acyl-CoA metabolic process, lipid metabolic process, fatty-acid transport, and triglyceride metabolic process (Figure 7A). KEGG pathway analysis revealed that PRF administration altered the expression of genes implicated in pathways such as PPAR signaling, retinol metabolism, fatty-acid degradation, metabolic pathways, linoleic-acid metabolism, and fatty-acid metabolism (Figure 7B). Transcriptome analyses indicated that PRF could exert its anti-obesity effect by regulating the genes associated with the fatty-acid metabolic process, lipid metabolic process, and retinol metabolism.

## 4. Discussion

Obesity-associated metabolic dysfunction is occurring as one of the most health-threatening diseases in the world [32]. Hence, there remains an insistent need for the development of efficient and safe therapeutic agents. Several reports have indicated that citrus PMFs could be appealing candidates due to their potent defensive effects against metabolic syndrome, as well as their safety in rodent experimental models [33,34]. Citrus PMFs, particularly nobiletin, tangeretin, and sinensetin, are abundant in the *Citrus* genus and exhibit various biological properties [15]. Recently, it was suggested that the beneficial effects of PMFs, which are groups of phenolic phytochemicals with poor bioavailability, are mostly exerted through the regulation of the gut microbiota [14,33]. Most of the research on PMFs has been conducted using fruits, and there is no study on PMFs isolated from citrus leaves. Here, we established an optimized and standardized procedure for preparing a PMF-rich fraction (PRF) from Jinkyool (*C. sunki*) leaves. The PRF was qualitatively and quantitatively characterized, and the compounds contained were clearly elucidated [20]. The PRF from leaves contained tangeretin (50.34 mg/g), nobiletin (15.81 mg/g), and sinensetin (1.10 mg/g) as the major constituents. The PRF from the leaves contained a three-times-higher content of PMFs compared to whole fruits that were harvested at the same time as the leaves. Jinkyool fruit is harvested once a year, but its leaves can be harvested year-round, and the leaves at all the harvesting times have a constant PMF content. Thus, we evaluated its potential as an anti-obesity dietary ingredient. There is no information on the average intake of PRF from citrus leaves. However, the animal studies using PMFs from citrus peels have shown beneficial effects at 30–120 mg/kg [14,15,35,36,37]. Based on this information, we implemented an administration dose of PRF at 50, 100, and 200 mg/kg of body weight.

Over 5 weeks of administration in HFD-induced obese mice, three doses of PRF exhibited an equal or even better obesity protective effect than a current anti-obesity drug, orlistat, whose mechanism involves inhibiting pancreatic lipase. We did not observe any obvious side effects of a high dose of PRF in HFD-induced obese mice. We noted no significant difference in the total food intake of mice in each group during the experimental period (*p* > 0.05), suggesting that PRF-induced weight loss involved a mechanism that was independent of hyperphagia as compared to the HFD group.

Adipose tissue can increase in size through hypertrophy or hyperplasia. The hyperplasia of adipose tissue is believed healthy and adaptive. However, the hypertrophy of adipocytes is related to an increase in the hypoxia experienced by these cells because of their massive expansion. Hypoxic adipose tissue increases the expression of pro-fibrotic genes and leads to tissue fibrosis. Hypoxic adipocytes occasionally experience necrosis, leading to infiltration by immune cells and tissue inflammation [38]. These factors work together to decrease adipose tissue function and consistently raise blood glucose and lipid levels. This might contribute to an earlier onset of metabolic disease and trigger toxic lipid deposition in other tissues [39,40,41]. PRF administration dose-dependently decreased the sizes of the epididymal adipocytes in a superior manner to orlistat in mice with HFD-induced obesity. This indicates that PRF might reduce HFD-induced weight gain by lowering the accumulation of triglycerides and the expansion of the adipose tissue. PRF effectively improved dyslipidemia and decreased the fasting blood glucose and insulin levels, resulting in improved glucose tolerance and insulin resistance in HFD-induced obese mice.

To explore the mechanism underlying the anti-obesity effect of PRF administration in HFD-induced obese mice, we analyzed the expression of lipid-catabolism-related proteins among the groups. PRF administration dramatically increased the phosphorylation of AMP-activated protein kinase (AMPK), acetyl-CoA carboxylase (ACC), and hormone-sensitive lipase (HSL) in the white adipose tissue of HFD-induced obese mice. These results suggest that PRF exerted its anti-obesity effects through the activation of AMPK. AMPK is a main regulator of cell and whole-body energy metabolism and is a crucial modulator of cell survival or death in response to pathological stress [42,43]. The activation of AMPK in adipocytes results in a decrease in triglyceride synthesis, lipogenesis, and inflammatory cytokine secretion, along with an increase in fatty-acid oxidation and GLUT4 translocation to cell membranes [42,43]. We found that the expression of HSL was significantly enhanced in the orlistat and PRF administration groups compared with the HFD group. HSL is a rate-limiting enzyme in the breakdown of triglycerides that are mainly involved in the hydrolysis of diglycerides to monoglycerides [44,45]. Thus, these results suggest that PRF exerted its anti-obesity effect by promoting both fatty-acid oxidation via the AMPK/ACC pathway and lipolysis via the PKA/HSL pathway in the WAT of HFD-induced obese mice. However, further studies on these mechanisms are needed.

PRF significantly decreased the serum GOT/GPT level and liver tissue weight in HFD-induced obese mice; thus, PRF might protect against the liver damage induced by an HFD. Consistent with this observation, the liver tissue of the HFD group had a typical fatty-liver shape. However, the number of lipid droplets was significantly reduced in the liver tissue of the groups administered PRF and orlistat. PRF administration increased the phosphorylation of AMPK/ACC in HFD-induced obese mice. Thus, it is suggested that PRF prevented fatty liver by both inhibiting de novo lipogenesis and promoting fatty-acid oxidation via AMPK activation. It is well known that AMPK plays a major role in maintaining the energy balance, including controlling lipid metabolism in the liver [46,47]. Indeed, our results suggest that AMPK activation could play a vital role in mediating the beneficial effects of PRF. Inflammatory factors, such as lipopolysaccharide (LPS) and tumor necrosis factor-α (TNF-α), reduce AMPK activity, whereas AMPK activation counteracts NF-κB signaling, which is a multifaceted protein complex that generates inflammatory factors in the fatty liver [48,49,50,51].

Thus, to further understand the mechanism underlying the anti-obesity effect of PRF in liver tissues, we analyzed differences in the liver transcriptome profiles between the HFD and PRF 50 groups by RNA-Seq. PRF administration mainly enriched GO terms associated with biological processes, such as the fatty-acid metabolic process, acyl-CoA metabolic process, lipid metabolic process, fatty-acid transport, and triglyceride metabolic process. The KEGG pathways affected by PRF administration in HFD-induced obese mice involved PPAR signaling, retinol metabolism, fatty-acid degradation, metabolic pathways, linoleic-acid metabolism, and fatty-acid metabolism. For example, the PPAR signaling pathway was shown to play a crucial role in energy homeostasis, glucose metabolism, and lipid metabolism [52]. PRF-enriched genes that are involved in the PPAR pathway include *CYP4A12A, FABP2, FABP5, ACSL1, EHHADH, FABP7, CYP4A10, ACAA1B, ACAA1B, CYP4A14, CD36*, and *CYP7A1*. The expression patterns of the fatty-acid uptake factors, such as CD36, FABPs, and CYP7A1, were differentially recovered to levels of the ND group by PRF administration in HFD-induced obese mice. Since these genes are involved in lipid deposition in the liver tissue [53,54,55,56,57,58], it is proposed that PRF prevents fatty liver by both suppressing lipogenesis and increasing fatty-acid oxidation in the liver. In this study, we did not validate the RNA-Seq data with qPCR; however, the changes in the transcriptome profiles were consistent with the difference in protein expression patterns between the PRF 50 and HFD groups. Taking into account all these results, we hypothesize that PRF might alleviate obesity-associated metabolic derangement by regulating relevant genes.

In conclusion, PRF suppressed body weight gain, white adipose tissue weight, hypertrophy of adipocytes, and fatty liver by regulating lipid-metabolism-related genes. PRF effectively improved dyslipidemia and insulin resistance in HFD-induced obese mice. These results demonstrate that the PRF might be a favorable therapeutic agent for metabolic disorders in terms of its efficacy and safety.

## Figures and Tables

**Table 1:** Effects of PRF on body weight food intake and organ weights in HFD-induced obese mice.

	ND	HFD	OR	PRF 50	PRF 100	PRF 200
Initial weight (g)	27.64 ± 1.26 ^a^	34.60 ± 6.20 ^b^	32.53 ± 3.78 ^b^	33.01 ± 4.61 ^b^	32.70 ± 3.11 ^b^	32.84 ± 2.83 ^b^
Final weight (g)	28.73 ± 0.30 ^a^	43.26 ± 5.68 ^c^	37.36 ± 3.98 ^b^	37.54 ± 5.12 ^b^	37.07 ± 3.56 ^b^	36.71 ± 4.66 ^b^
Weight gain (g)	1.26 ± 0.30 ^a^	8.66 ± 1.20 ^c^	4.83 ± 1.93 ^b^	4.53 ± 2.10 ^b^	4.37 ± 2.17 ^b^	3.87 ± 2.22 ^b^
Food intake (g/mouse/day)	4.08 ± 0.51 ^a^	2,76 ± 0.18 ^b^	3.05 ± 0.34 ^b^	2.89 ± 0.50 ^b^	2.61 ± 0.23 ^b^	2.74 ± 0.56 ^b^
Liver (g)	1.37 ± 0.08 ^bc^	1.49 ± 0.41 ^c^	1.09 ± 0.18 ^a^	1.36 ± 0.15 ^abc^	1.19 ± 0.17 ^ab^	1.25 ± 0.08 ^ab^
Epididymal fat (g)	0.67 ± 0.17 ^a^	2.55 ± 0.60 ^c^	2.11 ± 0.20 ^bc^	1.70 ± 0.41 ^b^	1.93 ± 0.41 ^b^	1.74 ± 0.77 ^b^
Kidney (g)	0.30 ± 0.03 ^a^	0.36 ± 0.02 ^b^	0.31 ± 0.04 ^a^	0.35 ± 0.03 ^b^	0.34 ± 0.02 ^b^	0.34 ± 0.03 ^b^
Spleen (g)	0.08 ± 0.01 ^a^	0.13 ± 0.02 ^b^	0.07 ± 0.02 ^a^	0.09 ± 0.01 ^a^	0.08 ± 0.02 ^a^	0.09 ± 0.03 ^a^

HFD-induced obese mice were orally administered the drug under an HFD for a further 5 weeks. ND, normal diet; HFD, high-fat diet; OR, HFD + 15.6 mg of orlistat; PRF 50, PRF 100, and PRF 200, HFD + 50, 100, and 200 mg of PRF, respectively. The results are expressed as the means ± SDs (*n* = 7). The values with different letters (a–c) are significantly different (*p* < 0.05) from each other according to one-way ANOVA and Duncan’s multiple-range test.

## Data Availability

The datasets generated for this study can be found in GEO, Accession NO. GSE193785.

## Supplementary Materials

The following supporting information can be downloaded at https://www.mdpi.com/article/10.3390/nu14040865/s1: Table S1. List of differentially expressed genes in HFD versus PRF 50 group.
