# Supplementary material for: Anti-Obesity Effects of Polymethoxyflavone-Rich Fraction from Jinkyool (Citrus sunki Hort. ex Tanaka) Leaf on Obese Mice Induced by High-Fat Diet
Source: Nutrients. 2022 Feb 18;14(4):865. doi: 10.3390/nu14040865 (PMC8878017; doi:10.3390/nu14040865)
Supplement: Supplementary file 1 [file nutrients-14-00865-s001.zip › nutrients-1582893-supplementary.pdf]

Table S1. List of differentially expressed genes (DEGs) in HFD versus PRF 50 Groups.

Gene symbol	Description	Fold Change
1810011O10Rik	RIKEN cDNA 1810011O10 gene	0.452
2010003K11Rik	RIKEN cDNA 2010003K11 gene	0.413
Abcc3	ATP-binding cassette, sub-family C (CFTR /MRP), member 3	0.494
Abhd6	abhydrolase domain containing 6	0.447
Acaa1b	acetyl-Coenzyme A acyltransferase 1B	0.301
Acot1	acyl-CoA thioesterase 1	0.186
Acot3	acyl-CoA thioesterase 3	0.054
Acot4	acyl-CoA thioesterase 4	0.213
Acsl1	acyl-CoA synthetase long-chain family member 1	0.469
Acss3	acyl-CoA synthetase short-chain family member 3	0.191
Aldh3a2	aldehyde dehydrogenase family 3, subfamily A2	0.368
Anxa2	annexin A2	0.471
Apoa4	apolipoprotein A-IV	0.230
Aqp8	aquaporin 8	0.460
C1qa	complement component 1, q subcomponent, alpha polypeptide	2.561
C1qb	complement component 1, q subcomponent, beta polypeptide	2.806
C1qc	complement component 1, q subcomponent, C chain	2.759
C6	complement component 6	3.971
C8a	complement component 8, alpha polypeptide	2.038
C8b	complement component 8, beta polypeptide	2.633
C9	complement component 9	2.002
Car3	carbonic anhydrase 3	0.385
Cd36	CD36 antigen	0.185
Cd52	CD52 antigen	2.238

Cd5l	CD5 antigen-like	2.966
Ces1d	carboxylesterase 1D	0.358
Ces1e	carboxylesterase 1E	0.450
Cfp	complement factor properdin	2.713
Cib3	calcium and integrin binding family member 3	6.993
Clec4f	C-type lectin domain family 4, member f	2.512
Crat	carnitine acetyltransferase	0.359
Csf1r	colony stimulating factor 1 receptor	2.601
Ctss	cathepsin S	2.328
Cxcl1	chemokine (C-X-C motif) ligand 1	3.012
Cyba	cytochrome b-245, alpha polypeptide	2.180
Cyp1a2	cytochrome P450, family 1, subfamily a, polypeptide 2	2.056
Cyp2b9	cytochrome P450, family 2, subfamily b, polypeptide 9	0.016
Cyp2c38	cytochrome P450, family 2, subfamily c, polypeptide 38	0.229
Cyp3a11	cytochrome P450, family 3, subfamily a, polypeptide 11	0.362
Cyp3a59	cytochrome P450, family 3, subfamily a, polypeptide 59	0.416
Cyp4a10	cytochrome P450, family 4, subfamily a, polypeptide 10	0.079
Cyp4a12a	cytochrome P450, family 4, subfamily a, polypeptide 12a	0.416
Cyp4a14	cytochrome P450, family 4, subfamily a, polypeptide 14	0.034
Cyp7a1	cytochrome P450, family 7, subfamily a, polypeptide 1	2.206
Cyp7b1	cytochrome P450, family 7, subfamily b, polypeptide 1	2.513
Dnajc12	DnaJ heat shock protein family (Hsp40) member C12	2.725
Egfr	epidermal growth factor receptor	3.365
Ehhadh	enoyl-Coenzyme A, hydratase /3-hydroxyacyl Coenzyme A dehydrogenase	0.484
Eif4ebp3	eukaryotic translation initiation factor 4E binding protein 3	2.243
Elovl5	ELOVL family member 5, elongation of long chain fatty acids (yeast)	0.456
Enho	energy homeostasis associated	5.905

Fabp2	fatty acid binding protein 2, intestinal	0.466
Fabp5	fatty acid binding protein 5, epidermal	2.733
Fabp7	fatty acid binding protein 7, brain	2.376
Fcer1g	Fc receptor, IgE, high affinity I, gamma polypeptide	2.224
Fcna	ficolin A	2.417
Fgl1	fibrinogen-like protein 1	3.077
Fitm1	fat storage-inducing transmembrane protein 1	0.439
G0s2	G0 /G1 switch gene 2	0.261
G6pc	glucose-6-phosphatase, catalytic	0.233
Gm4952	predicted gene 4952	0.488
Gngt2	guanine nucleotide binding protein (G protein), gamma transducing activity polypeptide 2	2.152
Gstp1	glutathione S-transferase, pi 1	4.350
Hamp2	hepcidin antimicrobial peptide 2	2.207
Hhex	hematopoietically expressed homeobox	2.049
Hmox1	heme oxygenase 1	2.882
Hp	haptoglobin	2.166
Hpx	hemopexin	2.303
Hsd3b5	hydroxy-delta-5-steroid dehydrogenase, 3 beta- and steroid delta-isomerase 5	6.441
Ifi27l2a	interferon, alpha-inducible protein 27 like 2A	2.697
Inhbe	inhibin beta-E	0.438
Lbp	lipopolysaccharide binding protein	2.237
Lcn2	lipocalin 2	13.831
Lgals1	lectin, galactose binding, soluble 1	0.251
Lgmn	legumain	2.117
Lrg1	leucine-rich alpha-2-glycoprotein 1	2.364
Lst1	leukocyte specific transcript 1	2.038

Ly6e	lymphocyte antigen 6 complex, locus E	2.269
Lyz2	lysozyme 2	2.518
Marco	macrophage receptor with collagenous structure	9.314
Me1	malic enzyme 1, NADP(+)-dependent, cytosolic	0.298
Mfsd2a	major facilitator superfamily domain containing 2A	0.369
Mme	membrane metallo endopeptidase	0.356
Moxd1	monooxygenase, DBH-like 1	21.842
Mpeg1	macrophage expressed gene 1	2.501
Mup20	major urinary protein 20	2.552
Orm2	orosomucoid 2	17.617
Paqr7	progestin and adipoQ receptor family member VII	0.469
Pex11a	peroxisomal biogenesis factor 11 alpha	0.292
Pklr	pyruvate kinase liver and red blood cell	0.442
Pla2g6	phospholipase A2, group VI	0.473
Plin2	.	0.351
Ppp1r3b	protein phosphatase 1, regulatory (inhibitor) subunit 3B	0.443
Rdh16	retinol dehydrogenase 16	0.407
Retsat	retinol saturase (all trans retinol 13,14 reductase)	0.394
Rgs16	regulator of G-protein signaling 16	0.464
Rnase4	ribonuclease, RNase A family 4	2.098
Saa1	serum amyloid A 1	12.793
Saa2	serum amyloid A 2	11.592
Sdc3	syndecan 3	2.754
Selenbp2	selenium binding protein 2	10.672
Serpina1e	serine (or cysteine) peptidase inhibitor, clade A, member 1E	15.511
Slc16a7	solute carrier family 16 (monocarboxylic acid transporters), member 7	0.459
Slc25a25	solute carrier family 25 (mitochondrial carrier, phosphate carrier), member 25	0.499

Slc25a47	solute carrier family 25, member 47	0.425
Slc3a1	solute carrier family 3, member 1	2.696
Steap4	STEAP family member 4	2.208
Tceal8	transcription elongation factor A (SII)-like 8	0.472
Tff3	trefoil factor 3, intestinal	19.510
Thrsp	thyroid hormone responsive	0.262
Tsc22d1	TSC22 domain family, member 1	0.442
Tsku	tsukushi, small leucine rich proteoglycan	0.444
Txnip	thioredoxin interacting protein	0.484
Tyrobp	TYRO protein tyrosine kinase binding protein	2.174
Vnn1	vanin 1	0.317
Vsig4	V-set and immunoglobulin domain containing 4	2.383
Wfdc17	WAP four-disulfide core domain 17	2.242
